# Brain Structural and Functional Imaging Findings in Medication-Overuse Headache

**DOI:** 10.3389/fneur.2019.01336

**Published:** 2020-01-28

**Authors:** Catherine D. Chong

**Affiliations:** Mayo Clinic, Phoenix, AZ, United States

**Keywords:** medication-overuse headache, migraine, structural and functional neuroimaging, pathophysiology, resting-state connectivity

## Abstract

This chapter overviews research neuroimaging findings of patients with medication-overuse headache (MOH). Results indicate; (i) correlations between neuropathology and medication-overuse; (ii) changes in brain morphology and cortical function; and (iii) brain recovery subsequent to withdrawal of medication that was overused. Results of this narrative review indicate exacerbated brain structural and functional changes in regions of the pain-matrix and in regions of the mesocortical-limbic circuit in patients with MOH compared to patients with migraine or compared to healthy controls. Modification of brain morphology as well as an association between brain recovery and medication withdrawal suggest that the MOH disease process involves state (brain modification) and trait-like (brain adaptation and recovery) neuromechanisms.

## Introduction

Medication-overuse headache (MOH) is a significant health concern affecting over 60 million people worldwide (1, 2). According to the International Headache Society (ICHD-3) guidelines (3), MOH is classified as a secondary headache disorder in patients who are overusing headache medication for treating a primary headache disorder. MOHs are classified as headaches occurring at least 15 days per month and overuse of headache medication is defined by the regular intake (>3 months) of medication for more than 10 days per month or more than 15 days per month, depending on the medication that is being overused. It is estimated that over 50% of patients in headache clinics who suffer from chronic forms of headache are overusing headache medication and develop MOH (4–7). The overuse of medication can lead to worsening of headache and the transition from episodic to chronic migraine. For patients with MOH, discontinuation of the medication that is being overused can result in a significant improvement of headache symptoms including a reduction in headache frequency (8–10); however, it is of note that the success rate of medication withdrawal varies widely among studies and likely depends on the medication that was being overused (11).

Although the neuropathology of MOH is still incompletely understood, research imaging of brain structure and function has helped our understanding of the involved neuromechanisms underlying MOH. This review will focus on studies that have used the following research neuroimaging techniques for evaluating brain structure, including T1-weighted MRI for interrogating gray and white matter structure, diffusion tensor imaging, for estimating integrity of white matter tracts, functional magnetic resonance imaging (fMRI) using resting-state paradigms to estimate the functional connectivity of brain networks, task-based fMRI to assess brain responses to specific stimuli, and positron emission tomography (PET) to evaluate the metabolic changes in glucose uptake in the brain. Studies are highlighted that have evaluated structural and functional imaging findings in patients with MOH compared to migraine patients without MOH, have assessed associations between brain changes and disease burden, and have examined brain mechanisms of recovery subsequent to medication withdrawal. Lastly, research neuroimaging differences between patients with MOH and patients with migraine are compared to neuroimaging differences between patients with post-traumatic headache relative to healthy controls in order to better understand neuropathological similarities and differences between MOH and post-traumatic headache, which are both discussed within this review series.

## Methods

For this review, PubMed was queried for English language articles using the following search terms: “Medication-overuse headache” and “magnetic resonance imaging” or “resting-state” or “diffusion tensor imaging” or “positron emission tomography.” Database search results were then limited to articles with relevance to the topic that were published between January 2000 and July 2019. Single-subject studies and studies that included <6 patients with MOH were excluded. Included in this review were 17 structural and functional imaging studies of patients with MOH (see Table 1).

**Table 1:** Medication overuse headache.

**References**	**Subject cohorts**	**Analysis**	**Findings**
**MEDICATION OVERUSE HEADACHE**			
Androulakis et al. (12)	• CM (*n* = 13) • HC (*n* = 19) • MOH (*n* = 16)	• rs-fMRI	MOH and CM vs. HC: central executive network alterations; less fc between right ventrolateral prefrontal cortex and left anterior thalamus, and less fc between left dorsal prefrontal cortex and dorsomedial prefrontal cortex. Alterations were more widespread in MOH vs. CM MOH vs. HC: DMN disruptions in MOH only
Androulakis et al. (13)	• CM (*n* = 13) • HC (*n* = 19) • MOH (*n* = 16)	• rs-fMRI	MOH and CM vs. HC: salience network disruptions in MOH and CM. MOH vs. CM: frontal-limbic network alterations in MOH between left DLPC and ventral striatum; left prefrontal cortex to right insula; left ventral striatum to left supplementary motor
Beckmann et al. (14)	• MOH^*^ (*n* = 27) • HC (*n* = 27) ^*^Before and 6 months after medication withdrawal	• VBM • TBSS	MOH (*before vs. after withdrawal)*: no difference in brain volume or diffusion measures (FA, RD) MOH vs. HC: no difference in brain volume or diffusion measures (FA, RD)
Bogdanov et al. (15)	• Mig during attack (*n* = 5) • Mig between attacks (*n* = 14) • HC (*n* = 24) • MOH (*n* = 7)	• Task-based fMRI*noxious warm and cold stimulation*	All subjects (main effect analysis): activation of pain-matrix when switching from warm to cold stimulation Mig during attack and MOH vs. HC: stronger motor cortex and superior temporal sulcus activation MOH vs. Mig during attack: stronger activation in premotor, supplementary motor, DMPC, ACC, primary somatosensory and lingual cortex
Chanraud et al. (16)	• MOH (*n* = 26) • EM (*n* = 23) • HC (*n* = 17)	• VBM • rs-fMRI	MOH vs. EM and HC: no difference in brain volume MOH vs. EM: less fc between left precuneus to frontal and parietal regions (right precuneus, right middle frontal, left inf. parietal and left cerebellum. Stronger left precuneus to temporal (right sup. temporal, right precentral, right fusiform and right hippocampus) region connectivity Clinical correlation—MOH only: negative correlation between migraine duration and frontal, precuneus and hippocampal volume; negative correlation between migraine duration and fc between precuneus and frontal regions; positive correlation between disease dependence and fc between precuneus and frontal regions; positive correlation between disease duration and left precuneus connectivity with bilateral temporal (inferior and superior), fusiform and right middle temporal gyrus positive relationship between numbers of pills taken and fc between left precuneus and hippocampus
Chen et al. (17)	• EM (*n* = 18) • HC (*n* = 32) • MOH (*n* = 37)	• rs-fMRI	MOH vs. EM: decreased fc density of right caudate and left insula and decreased fronto-temporal connectivity
Ferraro et al. (18)	• HC (*n* = 8) • CM (*n* = 8) • MOH^*^ (*n* = 8) ^*^Before and 6 months after withdrawal	• Task-based fMRI *Decision-making paradigm*	MOH vs. CM: less task-related activity in ventral tegmental area MOH vs. HC: less task-related activity in ventral tegmental area and stronger task-related activity in ventromedial prefrontal cortex MOH *(before vs. 6-months post-withdrawal)*: MOH prior to withdrawal vs. post-withdrawal: increased activity in ventromedial prefrontal cortex and posterior cingulate
Ferraro et al. (19)	• HC (*n* = 9) • MOH^*^ (*n* = 9) ^*^Before and 6 months after withdrawal	• Task-based fMRI *Noxious heat stimulation*	MOH (*at beginning of withdrawal*) vs. HC: reduced pain-related activation in somato-sensory cortex, inf. parietal lobule, supramarginal gyrus, middle cingulate and insula MOH (*at 6-month-post-withdrawal*) vs. HC: no difference in pain-related activity between groups
Fumal et al. (20)	• HC (*n* = 68) • MOH^*^ (*n* = 16) ^*^Before and 3 weeks after withdrawal	• FDG-PET *Glucose metabolism*	MOH *(before withdrawal*): hypometabolism in OFC, thalamus, anterior cingulate insula, and inf. parietal lobule. Hypermetabolism in cerebellar vermis MOH (*after withdrawal*): normalization of all regions except continued hypometabolism in OFC
Grazzi et al. (21)	• HC (*n* = 11) • MOH^*^ (*n* = 13) ^*^Before and 6 months after withdrawal	• Tasked-based fMRI *mechanical pain-induced stimulation*	MOH (*before withdrawal*) vs. HC: Less activation in regions of “lateral pain matrix” (inf. and superior parietal and supramarginal gyrus). Functional activation normalized after withdrawal
Krebs et al. (22)	• HC (*n* = 10) • MOH^*^ (*n* = 10) ^*^Before and 6–weeks after sphenopalatine ganglion (SPG) blocks	rs-fMRI	MOH (*before vs. after SPG blocks)*: Salience network: stronger fc between prefrontal cortex and insula, basal ganglia, motor and frontal cortex; stronger fc between temporal cortex and supramarginal gyrus and basal ganglia Executive network: stronger fc between prefrontal cortex and anterior thalamus and frontal cortex
Lai et al. (23)	• CM (*n* = 33) • HC (*n* = 33) • MOH (*n* = 33)	• VBM	MOH and CM vs. HC: less volume in precuneus, cerebellum and in temporal and occipital regions MOH vs. CM: less volume in OFC and middle occipital. More volume in in left parahippocampal and temporal pole region. Volume changes explained 31.1% variance of analgesic use frequency Clinical correlation in MOH: volume over OFC predicted treatment response
Mehnert et al. (24)	• HC (*n* = 18) • MOH^*^ (*n* = 18) ^*^Before and a minimum of 8 weeks after withdrawal	• VBM • rs-fMRI • Task-based fMRI*nociceptive stimulation*	MOH (*before withdrawal*) vs. HC: less volume in hippocampus, precuneus, inf. frontal gyrus, bilateral OFC, medial orbital gyrus MOH (*after withdrawal)* vs. HC: additional decreased volume in cuneus, superior temporal gyrus, putamen and cerebellum Clinical correlations: positive correlation between medial orbital gyrus volume in MOH before withdrawal and positive outcome (fewer headache days per month post-withdrawal Task-based activation during nociceptive stimulation MOH *before vs. after withdrawal*: stronger activation in left spinal trigeminal nucleus, right operculum and left posterior insula after withdrawal
Riederer et al. (25)	• MOH^*^ (*n* = 22) *11 responders 11-non-responders* ^*^Before and 3 months after withdrawal	• VBM	All MOH vs. HC: increased volume in PAG, ventral striatum, nucleus cuneiformis MOH -treatment responders: normalization in PAG, nucleus cuneiformis after withdrawal MOH-without treatment response: less OFC volume at baseline Clinical correlation: correlation between treatment response and OFC volume at baseline
Riederer et al. (26)	• HC (*n* = 29) • MOH (*n* = 29)	• SBM • Cortical thickness • gyrification	MOH vs. HC: less thickness in left prefrontal cortex. Higher gyrification over temporal, fusiform and right occipital pole Clinical correlation: higher gyrification over right occipital pole predicted poor treatment response
Schmidt-Wilcke et al. (27)	• MOH (*n* = 20) • Chronic tension-type headache (*n* = 20) • HC (*n* = 40)	• VBM	Chronic tension-type headache and MOH vs. HC: less volume in pain-processing regions in patients with Chronic tension-type headache compared to HC. No volume change in MOH compared to HC
Torta et al. (28)	• Migraine (*n* = 15) • MOH (*n* = 15)	• rs-fMRI	MOH vs. Migraine: machine-learning classification algorithms distinguished individual patients with MOH from patients with migraine with 75% accuracy based on the functional connectivity patterns of the nucleus accumbens

*ACC, anterior cingulate cortex; CM, chronic migraine; DMN, default-mode network; DMPC, dorso-medial prefrontal cortex; EM, episodic migraine; FA, fractional anisotropy; fc, functional connectivity; FDG-PET, F-fluoro-deoxyglucose positron emission tomography; HC, healthy controls; inf, inferior; Mig, migraine; MO, medication-overuse; MOH, medication-overuse headache; MwoA, migraine without aura; OFC, orbito-frontal cortex; PAG, periaqueductal gray; RD, radial diffusivity; SBM, surface-based morphometry; rs-fMRI, resting-state functional magnetic resonance imaging; TBSS, tract-based spatial statistics; VBM, voxel-based morphometry*.

## Results

### Brain Function and the Mesocortical-Limbic Circuit

In a pivotal PET study published in 2006, Fumal et al. (20) first reported hypometabolism in the orbitofrontal cortex, thalamus, anterior cingulate, insula, and the inferior parietal lobule as well as hypermetabolism in the cerebellar vermis in patients with MOH compared to healthy controls. Interestingly, 3 weeks after medication withdrawal, regional metabolic changes normalized, except for persistent hypometabolism in the orbitofrontal region. The orbitofrontal cortex is known to play a role in addictive disorders (29–31) and is part of the mesocortical-limbic circuit, involved in behaviors such as reward, motivation, pleasure, and sensation-seeking. Subsequent studies have further explored the connection between MOH and the functional connectivity and activation of relevant nodes of the mesocortical-limbic circuit including the extended limbic system (insula, amygdala, hippocampus, thalamus, and caudate) midbrain regions (ventral tegmental area and substantia nigra) and cortical frontal areas (prefrontal and orbitofrontal cortex).

These functional activation and resting-state functional connectivity studies have largely corroborated mesocortical-limbic dysregulation in patients with MOH. Ferraro et al. (18, 19) demonstrated less activity using a decision-making paradigm in the ventral tegmental region in MOH compared to chronic migraine and reduced pain-related activation in the middle cingulate and insula during heat pain stimulation in MOH compared to healthy controls. Torta et al. (28) showed evidence of nucleus accumbens to orbitofrontal functional dysregulation of the reward system that distinguished individual patients with MOH from migraineurs with a 75% accuracy. Two studies by Androulakis and colleagues suggest alterations in frontal-limbic networks and less functional connectivity within the left prefrontal cortex and between the right prefrontal cortex and the left anterior thalamus (12, 13).

Using functional connectivity density, a scale-free measurement of the brain's total number of connections, Chen et al. (17) found decreased functional connectivity density in the right caudate and the left insula in MOH compared to episodic migraine as well as weaker resting-state functional connectivity in fronto-temporal connectivity.

### Functional Alterations Within Regions of the Pain Matrix

In addition to mesocortical-limbic system dysfunction, patients with MOH have abnormalities within regions of the so-called “pain-matrix.” Results by Bogdanov et al. (15) indicate stronger activation of pain-processing regions (premotor, supplementary motor, dorsolateral prefrontal cortex, anterior cingulate, primary somatosensory) as well as stronger activation in the lingual region in patients with MOH relative to migraineurs using warm/cold noxious stimulation. Results by Grazzi et al. (21) show less activation using mechanical pain stimulation in the “lateral pain matrix” (inferior and superior parietal and supramarginal gyrus) in MOH relative to healthy controls and Chanraud and colleges (16) demonstrated decreased functional connectivity within regions of the default-mode system in MOH relative to episodic migraineurs.

### Functional Imaging: Before and After Medication Withdrawal

Multiple time-point studies in individual patients before and after withdrawal are undoubtedly the most informative for investigating how the brain changes or recovers subsequent to medication withdrawal and/or effective treatment. Krebs et al. (22) found improved functional connectivity within the salience and central executive networks after successful treatment using sphenopalatine ganglion (SPG) blocks in patients with MOH. SPG treatment in these patients resulted in decreased number of moderate to severe headache days per month, thus indicating that imbalance within both networks can be restored following successful treatment. Several studies have used nociceptive stimulation to investigate how the brain responds to pain before and after withdrawal. Mehnert et al. (24) found less activation in the left spinal trigeminal nucleus and the left posterior insula before withdrawal compared to after withdrawal and Grazzi et al. (21) demonstrated functional normalization in regions of the pain matrix subsequent to withdrawal.

### Brain Structure and the Orbitofrontal Cortex

Consistent with functional data, results of structural imaging reveal abnormalities within major regions of the mesocortical-limbic circuit in patients with MOH, including less orbitofrontal cortex volume and thickness (23, 24, 26). Results of several studies suggest that patients with better treatment response to medication withdrawal had more orbitofrontal volume at baseline, i.e., before withdrawal compared to patients who *did not* respond successfully to medication withdrawal (23, 25). Chanraud et al. found a negative correlation between frontal gray matter volume and migraine disease duration in patients with MOH (16) and between number of pills taken and frontal gray matter volume.

These results are intriguing and may indicate that the orbitofrontal cortex could be a marker for treatment response or indicative of poor outcome. In addition, male patients with MOH had less thickness over the left prefrontal region compared to females with MOH, raising the possibility of sex-related influences to developing MOH. These results are in line with other studies that have demonstrated sex-related differences in brain structure and function in patients with migraine (32–35) which is the topic of a comprehensive review in this series entitled “*Is there an MRI pattern that discriminates female from male migraine patients*” by Nasim Maleki and Xiao Androulakis.

Compared to healthy controls, Riederer et al. (26) found higher cortical gyrification over the temporal and occipital cortex in MOH (but not over the orbitofrontal cortex). In addition, patients with higher occipital pole gyrification had poorer response to medication withdrawal. As brain gyrification patterns remain relatively stable throughout the human lifespan, cortical gyrification is considered a proxy measurement of cortical development. These results are interesting and suggest a neurodevelopmental component to migraine disease chronification or genetic (trait-like) predisposition to a more severe disease type.

It is noteworthy that several studies did not find changes in brain structure in patients with MOH compared to healthy controls (16, 27), or in patients with MOH before compared to after withdrawal (14). Possibly, MOH induces more detectable changes in functional networks rather than brain macrostructure. Beckmann et al. (14) found no differences in brain volume or diffusion patterns in MOH patients before and after a 6-month withdrawal period. Interestingly, the patients included in this study were overusing medication an average of 4 years (including some patients who were overusing medication as long as 15 years). It is plausible that years of medication overuse decrease the ability of the brain to recover structurally, which might have contributed to the negative findings of this study.

## Discussion

Frequent medication overuse associates with exacerbated changes in regions of the pain matrix as well as with changes in structure and function of regions within mesocortical-limbic circuit. There is evidence of potential brain “traits” (higher occipital gyrification and less orbitofrontal cortex volume and thickness), which may contribute to the development of MOH or which may be a vulnerability factor for unsuccessful withdrawal.

Although the topic of “post-traumatic headache” (PTH) is part of another comprehensive review, entitled “Structural and Functional Brain Alterations in Post-traumatic Headache Attributed to Mild Traumatic Brain Injury,” the potential similarities and differences in neuropathology between primary headaches (migraine) and secondary (MOH and PTH) headaches are intriguing.

Migraine, MOH, and PTH show overlapping pathophysiology in regions associated with the cognitive-affective, sensory, and modulatory components of the “pain-matrix,” yet compared to migraine, patients with MOH and PTH have exacerbated changes within regions involved in multisensory integration (19, 21, 36, 37). MOH and PTH show *additional* neuropathology in brain systems that are not part of the pain matrix such as the visual cortex and specifically the lingual gyrus where hyperexcitability or sensitization is evident in both MOH and PTH (15, 36). Specifically, MOH has distinct structural and functional changes within regions of the mesocortical-limbic circuit implicated in addiction but also in reward, memory, motivation, and emotional response. Although there are currently no studies that have directly compared MOH to PTH, alterations within the mesocortical-limbic circuit and specifically the orbitofrontal cortex appear to be unique to MOH. There are some data suggesting rapid reorganization of cortical structure and function for both MOH and PTH. For example, several brain circuits demonstrate signs of brain normalization (or adaptation) weeks following successful withdrawal of patients with MOH. Similarly, patients with PTH at 1 week post-concussion show early alterations in brain structure. However, it is yet insufficiently understood whether these changes reflect mechanisms of neuronal repair, adaptation, or degeneration (38).

Although statements about the generalizability of neuroimaging findings in patients with MOH are premature, the number of studies published over the past few years has continued to increase our understanding of the neuropathology underlying MOH. Some of the current study discrepancies are likely due to between-study differences in sample sizes, the types of medications that were being overused (opiates vs. simple analgesics), the time frame over which overuse persisted (months vs. years), and whether patients experienced successful vs. unsuccessful withdrawal (headache relief vs. no headache relief, or relapse to MOH), which are important and often overlooked variables of the complex MOH disease mechanism (11) that will need to be better investigated and controlled for in future studies.

The most conclusive evidence of brain changes associated with MOH is derived from studies that have compared (i) patients with chronic migraine and MOH to patients with chronic migraine without MOH relative to cohorts of healthy controls; or assessed (ii) patients before and subsequent to medication withdrawal. Such studies are uniquely designed to interrogate and extract the disease pathology distinctive to medication overuse. Data from other studies that have compared MOH to healthy controls although informative are by design unable to extrapolate the pathology distinctive to medication overuse, as the migraine pathology and medication-overuse pathology are entangled.

## Conclusion

Compared to patients with migraine, patients with MOH have exacerbated changes in brain structure and function in regions of the pain-matrix and in areas of the mesocortical-limbic circuit. Some of the brain structural alterations (i.e., changes in brain gyrification patterns) could indicate “brain traits” that contribute to the development of MOH. Additionally, the relationship between brain changes and medication overuse (i.e., number of pills overused) or the association between brain recovery and discontinuation of medication overuse suggest “state-dependent” brain adaption patterns. In summary, these findings of state and trait-like changes suggest modification of brain structure and function in patients with MOH, some of which are likely reversible as patients recover from headache. Lastly, the neuropathological similarities of patients with MOH and post-traumatic headache may indicate common disease pathways that need to be further investigated.

## Author Contributions

The author confirms being the sole contributor of this work and has approved it for publication.

### Conflict of Interest

The author declares that the research was conducted in the absence of any commercial or financial relationships that could be construed as a potential conflict of interest.
